# Surface Microstructure Enhanced Cryogenic Infrared Light Emitting Diodes for Semiconductor Broadband Upconversion

**DOI:** 10.3390/nano14242039

**Published:** 2024-12-19

**Authors:** Peng Bai, Hanbin Wang, Rongrong Lv, Yi Wang, Yinqiao Li, Shangjie Han, Jiaxuan Cai, Ning Yang, Weidong Chu, Yan Xie, Meng Chen, Yingxin Wang, Ziran Zhao

**Affiliations:** 1 Institute of Applied Physics and Computational Mathematics, Beijing 100088, China; bai_peng@iapcm.ac.cn (P.B.); yiwang@mail.bnu.edu.cn (Y.W.); lyinqiao@bupt.edu.cn (Y.L.); shangjie.han@bupt.edu.cn (S.H.); caijiaxuan20@gscaep.ac.cn (J.C.); yang_ning@iapcm.ac.cn (N.Y.); 2 National Key Laboratory of Computational Physics, Beijing 100088, China; 3 Microsystem & Terahertz Research Center, China Academy of Engineering Physics (CAEP), Chengdu 610200, China; wanghanbin_mtrc@caep.cn; 4 Institute of Electronic Engineering, China Academy of Engineering Physics (CAEP), Mianyang 621900, China; 5 China Special Equipment Inspection and Research Institute, Beijing 100029, China; lvrong0908@126.com; 6 School of Physics and Astronomy, Beijing Normal University, Beijing 100875, China; 7 School of Science, Beijing University of Posts and Telecommunications, Beijing 100876, China; 8 Department of Engineering Physics, Tsinghua University, Beijing 100084, China; xie_yan@tsinghua.edu.cn (Y.X.); nk_chenmeng@163.com (M.C.); wangyingxin@tsinghua.edu.cn (Y.W.)

**Keywords:** cryogenic LEDs, electroluminescence efficiency, broadband upconversion, surface microstructure

## Abstract

Broadband upconversion has various applications in solar photovoltaic, infrared and terahertz detection imaging, and biomedicine. The low efficiency of the light-emitting diodes (LEDs) limits the broadband upconversion performance. In this paper, we propose to use surface microstructures to enhance the electroluminescence efficiency (ELE) of LEDs. Systematical investigations on the cryogenic-temperature performances of microstructure-coupled LEDs, including electroluminescence efficiency, luminescence spectrum, and recombination rate, have been carried out by elaborating their enhancement mechanism and light emitting characteristics both experimentally and theoretically. We have revealed that the reason for the nearly 35% ELE enhancement of the optimized structure under cryogenic temperature and weak injection current is the efficient carrier injection efficiency and the high recombination rate in the active region. We also compare studies of the surface luminescence uniformity of the optimized LED with that of the unoptimized device. This work gives a precise description, and explanation of the performance of the optimized microstructure coupled LED at low temperatures, providing important guidance and inspiration for the optimization of broadband upconverter in the cryogenic temperature region.

## 1. Introduction

Since its invention in 1962, GaAs-based LEDs have been widely used in various applications such as lighting, display, communication, greenhouse agriculture, and aerospace [1,2,3,4,5,6,7]. In recent years, with the development of infrared and terahertz upconversion technologies, this old topic has shown significant research value [8,9].

Among the many upconversion technologies, semiconductor photon frequency upconversion technology has shown great application potential due to its advantages in high compactness, fast response speed, good uniformity, flexible and adjustable spectrum, and free for readout integrated circuit (ROIC) [8]. In the past decades, researchers have developed various types of upconversion devices, such as inorganic upconversion, organic upconversion and inorganic-organic hybrid upconversion devices. Among them, traditional organic upconversion devices are integrated with organic detectors and organic light-emitting diodes, showing advantages such as high efficiency and low cost in the near-infrared region [10]. However, its environmental stability is a major challenge, and it is difficult to achieve upconversion in the mid-wave infrared and long-wave infrared regions. [11,12] Another type of organic up-conversion device is realized by colloidal quantum dot materials [13]. In principle, this type of device can achieve up-conversion from near-infrared to very long-wave infrared band, but how to integrate long-wave infrared upconversion devices still needs to be further explored, and the environmental stability, signal-to-noise ratio and sensitivity of the device are still somewhat worse than those of traditional semiconductor materials [14,15]. The organic-inorganic hybrid up-conversion devices that have been reported are all concentrated in the short-wave infrared region, and extending this approach to longer wavelength infrared is challenging [16,17]. In addition, the stacked structure of organic-inorganic materials also faces challenges in device stability, uniformity and carrier transport efficiency.

Up to now, up-conversion devices based on traditional inorganic semiconductor materials have the longest development time, mature fabrication technology and stable performance [18]. Researchers have successively achieved up-conversion in the near-infrared, mid-wave infrared, long-wave infrared and terahertz bands based on GaAs-based LEDs [19,20,21,22]. Since the LWIR up-conversion device that integrates GaAs-based LED with a quantum well-infrared photodetector (QWIP) was proposed in 1995, it has been developed for 8 years and successfully achieved long-wave infrared up-conversion thermal imaging with a noise equivalent temperature difference (NETD) of less than 60 mK in 2003 [21]. Using the same technical route, the THz band upconversion device was also reported in 2016 to successfully achieve THz upconversion pixelless imaging [22]. By integrating quantum cascade detectors with LEDs, mid-wave infrared upconversion can also be achieved under the action of an external bias [23]. What is even more surprising is that using wafer bonding technology, lattice-mismatched InGaAs detectors and InSb detectors can also be integrated with GaAs-based LEDs to successfully achieve near-infrared upconversion and mid-wave infrared upconversion, respectively [19,20]. However, it is not difficult to find that all of the above devices are narrow-band upconversion devices. The development of a broadband upconversion device that can simultaneously achieve near-infrared, mid-wave, long-wave, very long-wave infrared and terahertz band responses will greatly increase its application range. It can also reduce its payload while improving the compactness of the system.

An integrated HIWIP-LED (homojunction interface workfunction internal photoemission detector-light emitting diodes) device is a possible solution that can simultaneously achieve broadband responses covering the infrared and terahertz bands [24]. However, the operating temperature of HIWIP-LED devices is extremely low, and the hotspot problem of the LED is obvious. There is still a lot of room for optimization and improvement of the HIWIP-LED device. In 2021, the quantum ratchet upconversion devices proposed by Bai et al. may solve the problems of low operating temperature and serious light-emitting hot spots, but their upconversion efficiency is extremely low, and imaging cannot be achieved [25,26]. In fact, the low efficiency of LED is the fundamental reason that limits the efficiency of QRIP-LED devices. In addition, to achieve broadband upconversion with wavelengths extended to terahertz, its operating temperature often needs to be as low as liquid helium temperature. For example, the operating temperature of semiconductor upconversion devices such as HIWIP-LED and QWIP-LED that have been reported is ~4.2 K. Although another QRIP-LED is expected to achieve an increase in operating temperature, it will not be higher than liquid nitrogen temperature based on the working principle of the quantum ratchet. So, improving the efficiency and luminescence uniformity of LED ports at cryogenic temperatures is the prerequisite for achieving efficient broadband upconversion.

In this paper, we use an optimized LED structure to improve the quantum efficiency of LED devices. We also design a thick cap layer for the preparation of a micro-nano optical coupling structure to further improve the light extraction efficiency of the device. A microstructure surface is introduced to study the efficiency enhancement effect of the optical coupling structure on the planar structure in detail from both experimental and theoretical perspectives. At 10 K, the LED enhanced by the surface microstructure shows an increase in electroluminescence efficiency of nearly 35%. In addition, we compare the optimized LED with the unoptimized LED device in the previous QRIP-LED and discuss in depth the intrinsic reasons for the significant improvement in its electroluminescence efficiency, spectrum, and surface electroluminescence uniformity. The research results of this work will provide direct guidance for the realization of high-efficiency broadband upconversion devices and lay an important experimental and theoretical reference for the realization of large-format broadband up-conversion imaging.

## 2. Device Structure and Fabrication

**Figure 1:**
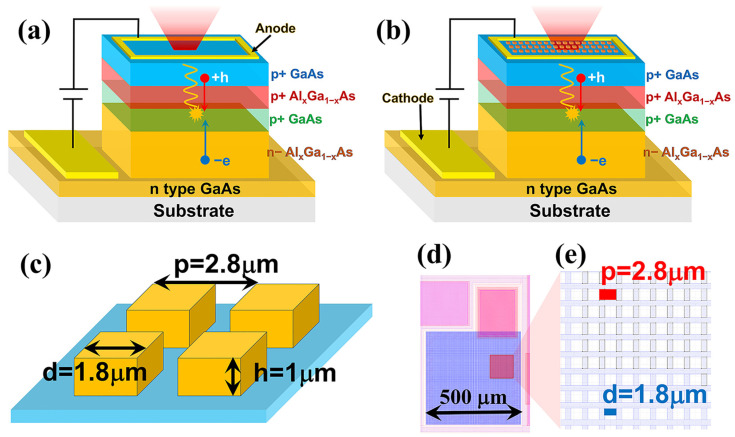
(**a**) The optimized LED device with a conversional planar structure. (**b**) The optimized LED with surface microstructure. (**c**) The detailed parameters of the micro mesas. (**d**) schematic diagram of the mask of the entire device. (**e**) A local pattern of the mask was used for surface microstructure preparation.

The optimized LED device with a conventional planar structure is shown in Figure 1a. The overall device is a GaAs/AlGaAs double heterojunction structure with an additional p-type GaAs cap layer.

The green area is the p-type doped GaAs active layer with a thickness of 400 nm, sandwiched between p-type AlGaAs and n-type AlGaAs. The detailed structural parameters are as follows: the epitaxial structure is grown on a semi-insulating GaAs(100) substrate. First a 50 nm GaAs buffer layer (with Si-doped to 2.5 × 10^18^ cm^−3^) is grown, followed by a 250 nm Al_0.45_Ga_0.55_As etch stop layer (with Si-doped to 2.5 × 10^18^ cm^−3^), above which is a 500 nm n-type doped GaAs bottom contact layer (with Si-doped to 2.5 × 10^18^ cm^−3^), and then three n-type AlGaAs barrier layers with different compositions and doping concentrations, from bottom to top are 350 nm Al_0.1_G_a0.9_As (with Si-doped to 2.5 × 10^18^ cm^−3^), 50 nm graded AlGaAs (with Al graded from 0.1 to 0.3 and with Si-doped to 2.5 × 10^18^ cm^−3^) and 100 nm of Al_0.3_Ga_0.7_As (with Si-doped to 1 × 10^18^ cm^−3^), followed by s 40 nm of intrinsic AlGaAs with Al component graded from 0.3 to 0.15, on top of which is a 400 nm p-type GaAs active layer (with Si-doped to 1 × 10^18^ cm^−3^). Growth continues with a completely symmetrical set of AlGaAs structures consisting of 40 nm intrinsic graded AlGaAs (with Al graded from 0.15 to 0.3), 100 nm p-type Al_0.3_Ga_0.7_As (with Be graded doped from 2 × 10^18^ cm^−3^ to 2 × 10^19^ cm^−3^), 50 nm graded AlGaAs (with Al graded from 0.3 to 0.1 and Be doped to 2.5 × 10^18^ cm^−3^), and 350 nm Al_0.1_Ga_0.9_As (with Be doped to 2 × 10^19^ cm^−3^), and finally a thick p-type GaAs cap layer (with Be doped to 2 × 10^19^ cm^−3^) is grown on top as the top contact layer and micro-structure etching layer. The top electrode is n-type (Pd 25 nm/Ge 75 nm/Ti 30 nm/Au 200 nm) metal, and the bottom electrode is p-type (Ti 20 nm/Pd 50 nm/Au 200 nm) metal.

The optimized LED with surface microstructure is shown in Figure 1b. Except that the surface is etched into a micro-mesa, the overall device is exactly the same as the standard mesa structure. The size, period, and height parameters of the micro-mesa we selected are typical structural parameters commonly used in up-conversion devices to achieve efficient quantum well detector light coupling. These parameters are given in detail in Figure 1c. The optical mask used for device preparation is shown in Figure 1d,e, where (d) is a schematic diagram of the mask of the entire device, and (e) is a local pattern of the mask used for surface microstructure preparation. The physical dimension of the overall device and micro mesa is also indicated in Figure 1d,e.

**Figure 2:**
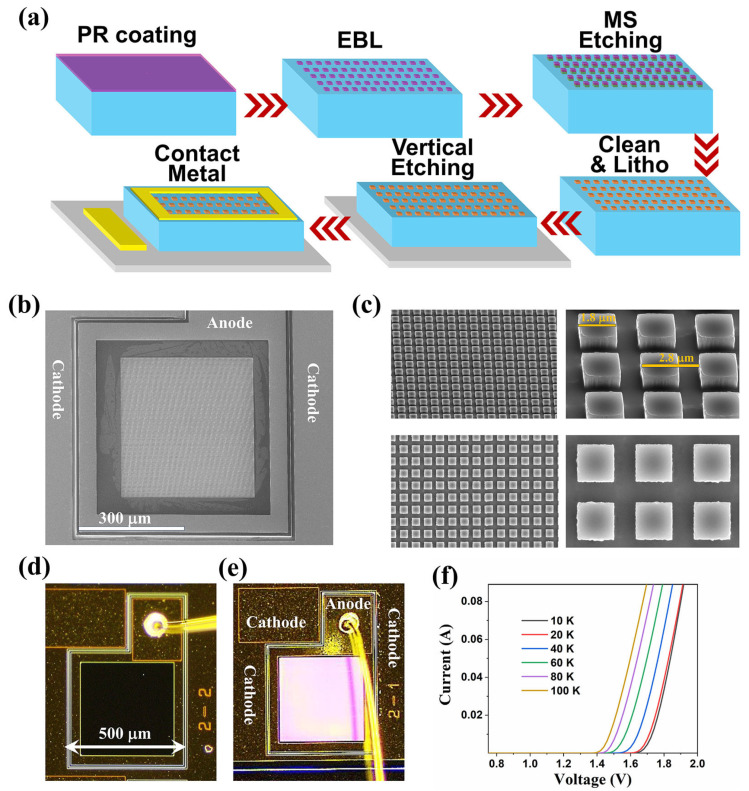
(**a**) The device preparation process flow, in which the arrow points represent the flow order. (**b**) The scanning electron microscope (SEM) image of the prepared microstructure device. (**c**) Magnified SEM image of the prepared microstructure device. (**d**) Optical microscope photograph of the prepared planar device without any microstructure. (**e**) Optical microscope photograph of the prepared device with microstructures. (**f**) I–V curves of the device at different temperatures.

The device preparation process flow chart is shown in Figure 2a. First, the LED epitaxial wafer is protected by photoresist (ZA5214) and then cleaved. The divided wafers are deeply cleaned with acetone, alcohol, and deionized water and then blown dry with N_2_. Next, the device is spin-coated with E-beam resist (PMMA 950K), and the surface microstructure square pattern with sides of 1.8 μm is exposed and developed using standard electron beam lithography technology. Then, Cl_2_ and BCl_3_ are used to etch the surface square microstructure, with an etching depth of about 1 μm, and the etching process is monitored by the laser interference oscillation method. After micro-pattern etching, the device is deeply cleaned again, and the photoresist is removed. Then, the mesa structure of the LED is etched using standard photolithography and dry etching processes, and the etching depth (about 3.3 μm) is also monitored in real-time using the laser interference oscillation method. After the LED mesa etching is completed, the sample is deeply cleaned again. After cleaning, the device is prepared for the top-ring and bottom-contact electrode metals. We use standard photolithography, electron beam evaporation and standard metal lift-off processes to prepare n-type (Pd 25 nm/Ge 75 nm/Ti 30 nm/Au 200 nm) and p-type (Ti 20 nm/Pd 50 nm/Au 200 nm) electrode metals. Dilute hydrochloric acid (HCl:H_2_O = 1:40) is used to treat the oxide layer before preparation, and rapid thermal annealing (RTA: 380 °C, 60 s) is performed after preparation to form a good ohmic contact. The fabricated LED devices are square mesa devices with a side length of 500 μm. The prepared samples are cleaved and packaged, and then performance characterization can be carried out.

The scanning electron microscope (SEM) image of the prepared microstructure device is shown in Figure 2b, where the optical window area is evenly covered with the surface microstructure. The detailed morphology of these microstructures is shown in Figure 2c, where the side length of the square micro-mesa is about 1.8 μm, and the height is about 1 μm. As shown in Figure 2d, it is an optical microscope photograph of the prepared device with a bonding Au wire. Due to the presence of the surface microstructure, under the reflection of the microscope light, the device surface shows a pink color different from the plane structure (as shown in Figure 2e). The device is a p-p-n-type double heterojunction LED structure. Therefore, it can only be turned on by applying a forward voltage to the p-n junction. After turning on, holes and electrons recombine in the active region to emit near-infrared light. The I–V curves of the device at different temperatures are shown in Figure 2f. The figure shows that when the temperature increases from 10 K to 100 K, the turn-on voltage of the device gradually decreases, which is consistent with the law of traditional light-emitting diodes. The main reason for the gradual decrease in the forward turn-on voltage with increasing temperature is that the band gap of the GaAs bulk material narrows with increasing temperature.

## 3. Results and Discussion

### 3.1. Electroluminescence Efficiency

**Figure 3:**
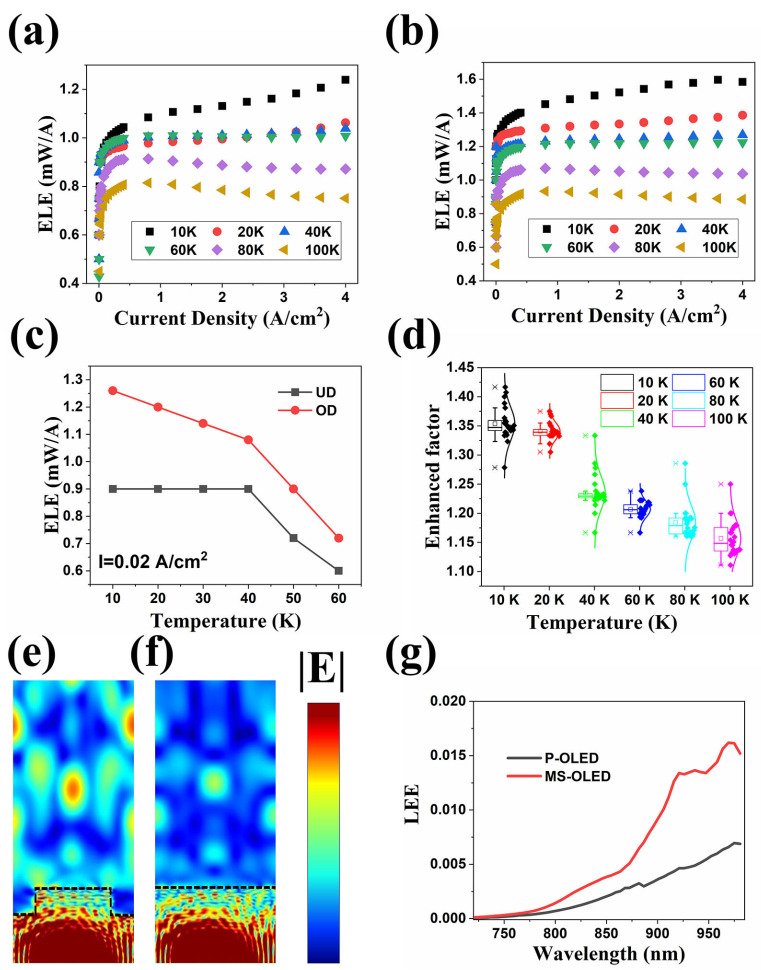
(**a**) Electroluminescence efficiency (ELE) of the OLED with surface microstructure. (**b**) Electroluminescence efficiency (ELE) of the OLED with planar structure. (**c**) Efficiency comparison of the two LED structures at different operating temperatures under the condition of weak current density injection of 0.02 A/cm^2^. (**d**) The statistical results of calculated enhanced factor. (**e**) The cross-sectional electric field intensities (|E|) distribution for the device with MS structure. (**f**) The cross-sectional electric field intensities (|E|) distribution for the planar device. (**g**) Calculated wavelength-dependent LEE of the two devices.

The device measurements were all carried out in a cryostat, and the gas pressure in the cryostat during the measurement was about 1 × 10^−5^ mbar. The electroluminescence efficiency (ELE) of the OLED with planar structure and surface microstructure is shown in Figure 3a,b. The ELE is obtained by dividing the obtained experimental electroluminescence power by the driving current of the LED device. The electroluminescence power on the device surface is measured by Thorlabs S130C (Thorlabs, NJ, USA) large area Si slim photodetector together with the PM100D Compact Power and Energy Meter Console (Thorlabs, NJ, USA). It should be pointed out that the measured power value at low temperatures needs to be calibrated. The calibration coefficient is achieved at room temperature. The calibration method is as follows: First, we put the light-receiving surface of the detector as close to the LED light-emitting surface as possible (with a distance of about 2 mm). Since the device area is about 0.25 mm^2^ and the detector area is 1 cm^2^, we believe that the detector collects almost all the LED surface EL power (P_out-RT_). Next, we put the LED in a cryostat and measured the device’s EL power (P_in-RT_) by placing the Si detector close to the quartz window of the cryostat at the same room temperature. The calibration coefficient was determined by comparing the P_out-RT_ and P_in-RT_. The power measured outside the cryostat at low temperature multiplied by the calibration coefficient is the actual EL power of the device at low temperature.

The measuring results show that compared with traditional P-OLEDs, MS-OLED devices have a significant improvement in ELE. When the temperature is 10 K, and the injection current is 4 A/cm^2^, the ELE of P-OLED is about 1.2 mW/A, but the ELE of MS-OLED is close to 1.6 mW/A, which is about 1/3 higher than the efficiency. When the temperature is 100 K, and the injection current is 4 A/cm^2^, the ELE of P-OLED is about 0.7 mW/A, but the ELE of MS-OLED is close to 0.85 mW/A, which is still more than 10% higher. Figure 3c shows the efficiency comparison of the two LED structures at different operating temperatures under the condition of weak current density injection of 0.02 A/cm^2^. We can find that even at a weak injection current density, MS-OLED has a certain efficiency improvement compared to P-OLED at different temperatures. To further confirm the efficiency enhancement effect of microstructure, we calculated the enhancement factor at various operating temperatures (10 K–100 K) and different injection current densities (0.004 A/cm^2^–4 A/cm^2^), which is defined as the ratio of ELEs at the same temperature and injection current density. The statistical results of the calculated enhanced factor are shown in Figure 3d. Inspection of the figure reveals that MS-OLED devices have obvious efficiency enhancement effects in all measuring temperature ranges.

However, as the temperature increases, the enhancement effect weakens. When the temperature increases from 10 K to 100 K, the average value of the enhanced factor decreases from 1.35 to 1.15. We attribute this phenomenon to the increase in non-radiative recombination rates such as SRH recombination and surface recombination. As the SRH recombination law is known, the gradual increase in temperature will increase the SRH recombination rate. Due to the etching of the micro-mesa structure, the specific surface area of the MS-OLED device is larger than that of the P-OLED device. The increase in temperature will increase the activity of SRH recombination centers and surface recombination centers, thereby reducing effective radiative recombination, resulting in a decrease in the overall internal quantum efficiency and microstructure enhancement efficiency. In order to understand the microstructure enhancement effect more intuitively, we carried out the 3D optical simulations with the finite difference time domain (FDTD) method to calculate the LEE for devices with planar structure and MS structure. The cross-sectional electric field intensities (|E|) distribution for the two structures are displayed in Figure 3e,f. The wavelength of the dipole source is set at 820 nm, and the periodic boundary condition is used. It is not difficult to find that most of the light is confined inside the planar device in Figure 3e. In the MS-OLED, apparently, the LEE of the device is improved, and more photons can escape from the active region of the LED. We calculated the wavelength-dependent LEE of the two devices, and the results presented in Figure 3g show obvious enhancement when the emitting wavelength is longer than 750 nm. At the wavelength of 820 nm, the LEE of the MS-OLED is about twice that of P-OLED. The difference between the theoretical results and the experiment may be caused by the surface leakage current since the device surface has not been passivated. Moreover, the bandgap of the thick GaAs cap layer matches with the emitting wavelength of the active layer, which may result in severe reabsorption of the photons and decrease the enhanced effect.

### 3.2. Electroluminescence Spectrum

**Figure 4:**
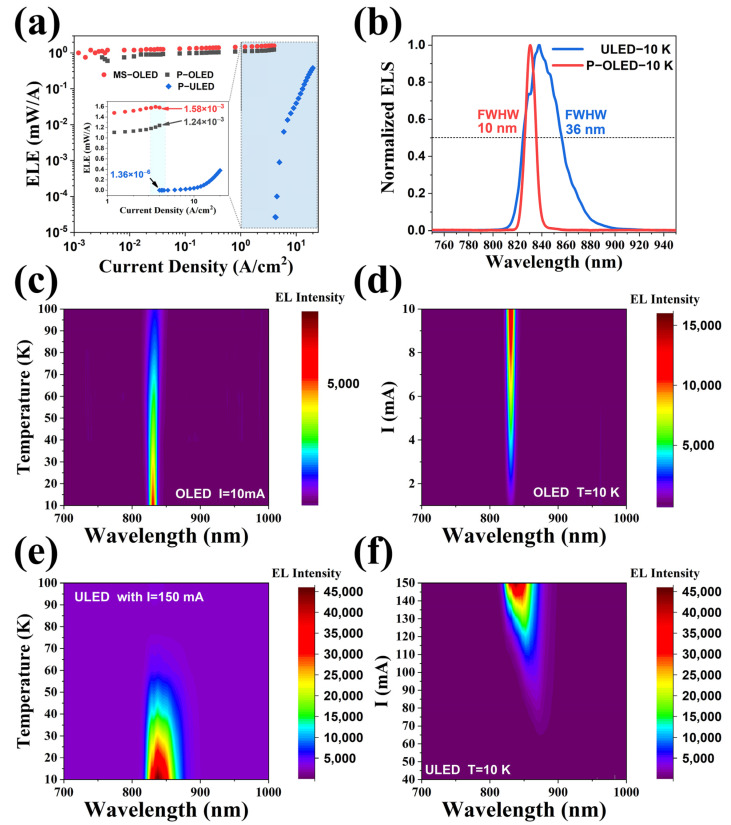
(**a**) The ELE of the three LED devices (MS-OLED, P-OLED&P-ULED), in which the dotted lines show the zoom area. (**b**) Emission spectra of the two devices (P-OLED&P-ULED). (**c**) Emission spectra of the P-OLED at different temperatures with an injection current of 10 mA. (**d**) Emission spectra of the P-OLED with different injection currents at 10 K. (**e**) Emission spectra of the ULED at different temperatures with an injection current of 150 mA. (**f**) Emission spectra of the ULED with different injection currents at 10 K.

In order to demonstrate the superiority of the optimized LED devices in the up-conversion devices, we compared the performance of P-OLED and MS-OLED with the LEDs in the previous broadband up-conversion devices. Figure 4a shows the ELE of the three LED devices. It is not difficult to find that the optimized LED has higher device efficiency. The ELE of both MS-OLED and P-OLED is significantly higher than that of ULED. In the measured current density range, the efficiency of OLED generally reaches more than 5 × 10^−4^ W/A, while the ELE of ULED is less than 4 × 10^−4^ W/A even at a very large injection current (20 A/cm^−2^). In addition, the turn-on current of OLED is more than three orders of magnitude smaller than that of ULED, and it shows high ELE even under weak current. The ELE difference is particularly obvious in the enlarged illustration that was inserted. When the injection current is 4 A/cm^−2^, the efficiency of OLED is five orders of magnitude higher than ULED. OLED and ULED also show obvious differences in the EL spectra.

Figure 4b shows the emission spectra of the two devices. OLED shows a nearly complete Lorentz line shape at 10 K, which meets the characteristics of standard LEDs. The line shape of ULED is not particularly smooth, even at 10 K, which hardly meets the characteristics of the Lorentz line shape. It can also be found that OLED has a smaller Full Width at Hall Maximum (FWHM) than ULED. The above phenomenon is particularly evident in the mapping results of Figure 4c–f. Inspection of the figures reveals that OLED shows good monochromaticity and a small FWHM at a specific temperature or a specific injection current. Moreover, a high emission spectrum intensity can be observed at 100 K with an injection current of 1 mA.

On the contrary, ULED shows poor monochromaticity under specific temperatures or specific injection currents, and the FWHM value is large. Moreover, it can only emit light effectively under high injection current (>40 mA), and the intensity of the emission spectrum drops rapidly after the temperature rises. One possible reason is the poor quality of the materials. The lack of long-range order in the atomic arrangement of local materials in the active region may introduce lots of dislocation and defects, which leads to the appearance of band tail states, resulting in poor monochromaticity and low ELE. In addition to the low internal quantum efficiency of unoptimized devices, internal defects may also cause the carriers involved in radiative recombination to not follow the standard Boltzmann distribution. The result is that the EL intensity fluctuates significantly with the wavelength (or energy). This fluctuation causes the discontinuity of the EL spectrum, which behaves in an irregular and unsmooth spectral line shape, and it is difficult to approximate it with the Lorentz function. The optimized device has a high internal quantum efficiency, and the carriers involved in the luminescence are more composite with the standard Boltzmann distribution, with high recombination efficiency and small recombination rate fluctuations. Therefore, the EL spectrum of the optimized device is more regular and smoother.

### 3.3. Band Structure

**Figure 5:**
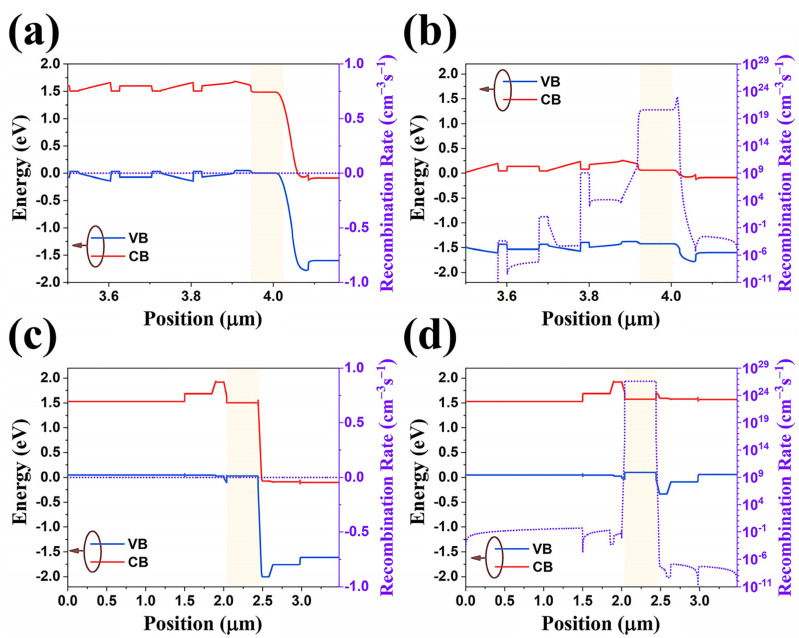
The device band structure of the QRIP-LED device at 0 V bias (**a**) and 2 V bias (**b**). The device band structure diagrams at the bias of 0 V bias (**c**) and 1.67 V bias (**d**). The yellow area marks the active area of LED.

The device structure of the above-presented ULED is derived from our previous QRIP-LED device. In order to have a deeper understanding of the fundamental reason for the performance enhancement of the optimized device, we calculated the band structure of the QRIP-LED device and the recombination rate inside the device. In the band structure calculation process, the ratio of conduction- and valence-band-offsets is assumed to be 6:4. Figure 5a,b shows the device band structure of the QRIP-LED device at 0 V bias and 2 V bias, respectively. In order to have a more intuitive inspection of the performance of the ULED part, we have given a magnified picture of the ULED part. We can find that at 0 V bias, the device is not turned on, so the recombination rate in each functional layer is 0. When the ULED is turned on, we can find that the recombination rate in the active layer area of the device can be close to 3 × 10^20^ cm^−3^s^−1^. In comparison, we applied a bias voltage of about 1.67 V in OLED to ensure the same electric field intensity as in the ULED device. The device band structure diagrams at bias of 0 V and 1.67 V are shown in Figure 5c,d, respectively. Similar to ULED, under 0 V bias, the LED is not turned on, so the recombination rate in each functional layer is 0. However, when the energy band becomes flat, and the device is turned on, we find that the recombination rate in the active region of the device can reach about 3 × 10^26^ cm^−3^s^−1^. This value is 6 orders of magnitude higher than that of the ULED device, which is the fundamental reason for the performance improvement of the device shown in Figure 4a.

A detailed analysis of the device band structure shows that although the doping concentration of the device active region is the same (1 × 10^18^ cm^−3^), the thickness of the ULED active region is only one-fourth of the thickness of the OLED active region. In theory, a thinner active region can give full play to the advantages of double heterojunction LEDs and improve the internal quantum efficiency of LEDs. However, if the active region is too thin, carriers may flow over the active region, thereby decreasing injection efficiency. The poor electron-hole injection efficiency will directly affect the radiative recombination efficiency of the active region. In addition, we can find that the energy of potential barriers on both sides of the ULED active region is low, and the restriction effect on the carriers in the active region is poor, resulting in a relatively low radiative recombination rate of the device. The high potential barriers on both sides of the OLED active region can better constrain carriers and increase the carrier concentration in the active region. The gradient barrier structure design in OLED also improves the carrier injection efficiency. Under the premise of thick active layer thickness, high heterojunction barrier and gradient barrier design, the ELE of OLED is significantly better than that of ULED. However, it must be pointed out that, in theory, the integral recombination efficiency of the OLED active region is more than 6 orders of magnitude higher than that of ULED, which is somewhat different from the experimental results. We attribute this phenomenon to the design of the thick, heavily doped cap layer structure in the OLED device. Although this structure provides sufficient space for the processing design of the microstructure, it also causes relatively serious reabsorption and sacrifices some ELE of LED devices.

### 3.4. Electroluminescence Uniformity

**Figure 6:**
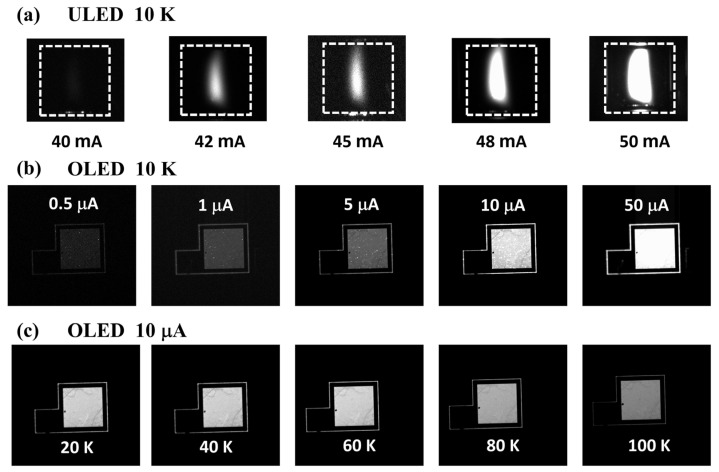
(**a**) Electroluminescence image of ULED at 10 K captured and recorded by CCD. (**b**) Electroluminescence image of P-OLED at 10 K. (**c**) Electroluminescence image of OLED at different temperatures with an injection current of 10 μA.

The surface luminescence uniformity of the LED device was characterized and captured by the Andor CCD camera (iKon-M 934 BR-DD) (Oxford Instruments, Oxfordshire, UK), and the results are shown in Figure 6. It can be found that the ULED device in Figure 6a begins to emit obvious light at a current greater than 40 mA (4 A/cm^2^). Moreover, the light emission of ULED is extremely uneven. Only a small area in the center of the device can emit light.

Even if the injection current increases to more than 50 mA, the entire device surface cannot achieve the whole surface uniform emission. This is another manifestation of low LED efficiency. On the contrary, Figure 6b shows that OLED can emit light even at a very weak injection current of less than 1 mA, and the light emission is extremely uniform. In addition, we also found that under weak current conditions (<5 mA), some small light-emitting hot spots appeared in the device. However, it is worth noting that these hot spots are not particularly bright, and the intensity is not significantly higher than the intensity of the entire device’s surface emission. When the injection current is 10 mA, the hot spotlight emission is basically at the same order of magnitude as the background light intensity, and the impact is almost eliminated. In Figure 6c, we found that as the temperature increases, the luminous intensity of the device gradually decreases, which is consistent with the previous efficiency and spectrum results.

## 4. Conclusions

In conclusion, we proposed a surface microstructure coupled LED to improve the performance. We grew a thick GaAs cap layer on the surface of the optimized LED device for microstructure etching. A detailed process flow chart for device preparation is also given. We studied in detail the enhancement of the electroluminescence efficiency of LED devices with the surface microstructures. The results show that the electroluminescence efficiency of devices with surface microstructures is 35% higher than that of planar structures when the temperature is below 20 K. The microstructure enhancement effect will be somewhat weakened due to the enhancement of non-radiative recombination of the device when the temperature rises. This problem can be optimized by introducing a subsequent passivation process. We also conducted an in-depth comparative study on the performance of the optimized device with ULED at cryogenic temperatures. The results show that OLED devices have a significant improvement in electroluminescent efficiency, emission spectrum, and luminescent uniformity. Among them, at 4 A/cm^2^, the ELE of OLED is 5 orders of magnitude higher than that of ULED and has a more regular and smooth luminescent spectrum line shape. At the same time, the minimum turn-on current of OLED is 3 orders of magnitude smaller than that of ULED. Finally, we compared the surface luminescence of OLED and ULED devices. The experimental results show that the surface luminescence of OLED is more uniform without overly bright luminescent hot spots, which is an ideal candidate for upconversion devices. This work lays a solid foundation for the realization of high-performance broadband upconversion devices and pixelless imaging.

## Data Availability

The data presented in this study are available on request from the corresponding authors.

## References

[1] Yam F.K., Hassan Z. (2005). Innovative advances in LED technology. Microelectron. J..

[2] Wang Y., Alonso J.M., Ruan X. (2017). A review of LED drivers and related technologies. IEEE Trans. Ind. Electron..

[3] Wu T., Sher C.-W., Lin Y., Lee C.-F., Liang S., Lu Y., Huang Chen S.-W., Guo W., Kuo H.-C., Chen Z. (2018). Mini-LED and micro-LED: Promising candidates for the next generation display technology. Appl. Sci..

[4] Karunatilaka D., Zafar F., Kalavally V., Parthiban R. (2015). LED based indoor visible light communications: State of the art. IEEE Commun. Surv. Tutor..

[5] Huang Y., Hsiang E.L., Deng M.Y., Wu S.T. (2020). Mini-LED, Micro-LED and OLED displays: Present status and future perspectives. Light Sci. Appl..

[6] Paradiso R., Proietti S. (2022). Light-quality manipulation to control plant growth and photomorphogenesis in greenhouse horticulture: The state of the art and the opportunities of modern LED systems. J. Plant Growth Regul..

[7] Petrick J.T. (2002). High-brightness LEDs in aerospace applications. Solid State Lighting II.

[8] Luo H., Ban D., Liu H.C., Wasilewski Z.R., Buchanan M. (2006). Optical upconverter with integrated heterojunction phototransistor and light-emitting diode. Appl. Phys. Lett..

[9] Ban D., Luo H., Liu H.C., Wasilewski Z.R., Buchanan M. (2005). Pixelless 1.5-μm up-conversion imaging device fabricated by wafer fusion. IEEE Photonics Technol. Lett..

[10] Hu X., Xiao G., Li Y., Wu S.E., Chen Q., Li N., Sui X. (2023). Infrared-Light Visualization by Organic Upconversion Devices. ACS Appl. Electron. Mater..

[11] Fu C., Mu G., Weng K., Tang X. (2024). Advances in Organic Upconversion Devices. Photonics.

[12] Rao T., Hao Q., Mu G., Qin T., Tan Y., Zhao P., Kong D., Chen M., Tang X. (2023). Large-scale fabrication of CMOS-compatible silicon-OLED heterojunctions enabled infrared upconverters. APL Photonics.

[13] Zhou W., Shang Y., de Arquer F.P.G., Xu K., Wang R., Luo S., Xiao X., Zhou X., Huang R., Sargent E.H. (2020). Solution-processed upconversion photodetectors based on quantum dots. Nat. Electron..

[14] Xue X., Hao Q., Chen M. (2024). Very long wave infrared quantum dot photodetector up to 18 μm. Light Sci. Appl..

[15] Tang X., Ackerman M.M., Chen M., Guyot-Sionnest P. (2019). Dual-band infrared imaging using stacked colloidal quantum dot photodiodes. Nat. Photonics.

[16] Chen J., Tao J., Ban D., Helander M.G., Wang Z., Qiu J., Lu Z. (2012). Hybrid Organic/Inorganic Optical Up-Converter for Pixel-Less Near-Infrared Imaging. Adv. Mater..

[17] Chu X., Guan M., Li L., Zhang Y., Zhang F., Li Y., Zhu Z., Wang B., Zeng Y. (2012). Improved efficiency of organic/inorganic hybrid near-infrared light upconverter by device optimization. ACS Appl. Mater. Interfaces.

[18] Yang Y., Zhang Y.H., Shen W.Z., Liu H.C. (2011). Semiconductor infrared up-conversion devices. Prog. Quantum Electron..

[19] Luo H., Ban D., Liu H.C., SpringThorpe A.J., Wasilewski Z.R., Buchanan M., Glew R. (2004). 1.5 μm to 0.87 μm optical upconversion using wafer fusion technology. J. Vac. Sci. Technol. A.

[20] Ban D., Luo H., Liu H.C., Wasilewski Z.R., Paltiel Y., Raizman A., Sher A. (2005). Midinfrared optical upconverter. Appl. Phys. Lett..

[21] Dupont E., Byloos M., Oogarah T., Buchanan M., Liu H.C. (2005). Optimization of quantum-well infrared detectors integrated with light-emitting diodes. Infrared Phys. Technol..

[22] Fu Z.L., Gu L.L., Guo X.G., Tan Z.Y., Wan W.J., Zhou T., Shao D.X., Zhang R., Cao J.C. (2016). Frequency up-conversion photon-type terahertz imager. Sci. Rep..

[23] Wang L., Hao Z.B., Luo Y., Kang J.B., Wang L., Xiong B., Sun C.Z., Wang J., Han Y.J., Li H.T. (2015). Semiconductor up-converter based on cascade carrier transport for infrared detection/imaging. Appl. Phys. Lett..

[24] Bai P., Zhang Y., Wang T., Fu Z., Shao D., Li Z., Wan W., Li H., Cao J., Guo X. (2019). Broadband THz to NIR up-converter for photon-type THz imaging. Nat. Commun..

[25] Bai P., Li X., Yang N., Chu W., Bai X., Huang S., Zhang Y., Shen W., Fu Z., Shao D. (2022). Broadband and photovoltaic THz/IR response in the GaAs-based ratchet photodetector. Sci. Adv..

[26] Bai P., Yang N., Chu W., Zhang Y., Shen W., Fu Z., Shao D., Zhou K., Tan Z., Li H. (2021). Ultra-broadband THz/IR upconversion and photovoltaic response in semiconductor ratchet-based upconverter. Appl. Phys. Lett..

